# Folic acid-targeted polymeric complex imprinted with doxorubicin and quercetin for controlled drug delivery in cancer therapy

**DOI:** 10.55730/1300-0527.3905

**Published:** 2026-06-02

**Authors:** Ceren TÜRKCAN, Emirhan İŞERİ

**Affiliations:** 1 Department of Biomedical Engineering, Faculty of Engineering, İstanbul Arel University, İstanbul, Turkiye; 2 Department of Biomedical Engineering, Postgraduate Education Institute, İstanbul University-Cerrahpaşa, İstanbul, Turkiye

**Keywords:** Doxorubicin, quercetin, controlled drug release, molecular imprinting, targeted therapy, polymeric complex

## Abstract

Cancer therapy is often limited by the systemic toxicity and dose-dependent adverse effects of conventional chemotherapeutic agents such as doxorubicin (DOX). In this study, a folic acid-functionalized molecularly imprinted polymeric complex based on poly(HEMA) crosslinked with ethylene glycol dimethacrylate was synthesized for the controlled release of DOX in combination with quercetin (QUE), a flavonoid known for its antioxidant and chemosensitizing properties. The aim of this work was to develop and physicochemically characterize a preliminary targeted drug delivery platform at a material-design level. The polymeric complex was synthesized via free-radical polymerization following a DOX–QUE precomplex formation step. Structural characterization was performed using Fourier transform infrared (FTIR) and scanning electron microscopy (SEM) analyses. SEM micrographs revealed nanoscale spherical and partially aggregated polymeric domains, while FTIR spectra confirmed the successful incorporation of DOX, QUE, and folic acid into the poly(HEMA) matrix. In vitro release studies were conducted in 0.1 M acetate buffer at pH 5.5 to simulate mildly acidic tumor-relevant conditions. Within 180 min, measurable release was observed, with 0.0011 mg of DOX and 0.0978 mg of QUE released from the system. Thus, this study demonstrates the structural feasibility of a folic acid-functionalized imprinted polymer system capable of controlled release under acidic conditions, presenting a material-level physicochemical evaluation of a dual-drug imprinted polymeric platform. Biological validation, including cytotoxicity assays and receptor-mediated uptake studies, is not addressed in this study and remains necessary for comprehensive therapeutic assessment.

## 1. Introduction

Cancer remains one of the leading causes of death worldwide despite significant advancements in early detection and treatment strategies. Among the therapeutic options, chemotherapy continues to play a central role. However, the use of chemotherapeutic agents like doxorubicin (DOX) is often limited by severe side effects and the development of drug resistance [1–3]. These challenges underscore the need for more sophisticated drug delivery systems that can increase drug specificity and reduce toxicity.

In recent years, the integration of biocompatible polymers, molecular imprinting techniques, and targeted delivery strategies has emerged as a promising approach to overcome these limitations [4]. Molecularly imprinted polymers offer a robust platform for the selective recognition and release of therapeutic agents [5]. When they are combined with bioactive compounds such as quercetin (QUE), a flavonoid with antioxidant and chemosensitizing properties, the therapeutic potential of such systems can be further enhanced [6,7].

Moreover, folic acid has gained attention as a targeting ligand due to its high affinity for folate receptors, which are overexpressed in various cancer cells [8]. By decorating polymeric carriers with folic acid, selective uptake by cancer cells can be achieved, minimizing off-target effects and improving treatment outcomes.

In recent years, the development of dual-drug delivery systems has gained significant attention due to their potential to enhance therapeutic efficacy through synergistic effects. Conventional drug delivery systems often suffer from limitations such as nonspecific distribution and uncontrolled release. Molecularly imprinted polymers offer a promising approach by providing selective binding sites tailored to target molecules. In this context, the incorporation of folic acid as a targeting ligand is particularly advantageous, as folate receptors are overexpressed in various cancer cells. Therefore, the combination of dual-drug imprinting and folic acid functionalization facilitates a potential strategy for improving targeted and controlled drug delivery systems.

This study presents the design and evaluation of a polymeric complex imprinted with DOX and QUE and functionalized with folic acid. The aim was to construct a dual-drug delivery system capable of controlled release and receptor-specific targeting, representing a material-based strategy for controlled and receptor-associated drug delivery research.

The primary objective of this study was to synthesize and characterize a folic acid-functionalized poly(HEMA)-based dual-drug delivery platform imprinted with DOX and QUE. The developed polymeric system was designed to evaluate structural integrity, surface morphology, and controlled release behavior under mildly acidic conditions. Accordingly, the study was constructed with a clearly defined material-based objective focusing on the development and characterization of a targeted polymeric drug delivery platform.

## 2. Materials and methods

### 2.1. Materials

Doxorubicin hydrochloride (Cat. No. D1515, Sigma-Aldrich, St. Louis, MO, USA), quercetin (≥95% purity; Cat. No. Q4951, Sigma-Aldrich), folic acid (≥97% purity; Cat. No. F7876, Sigma-Aldrich), 2-hydroxyethyl methacrylate (HEMA; Cat. No. 477028, Sigma-Aldrich), ethylene glycol dimethacrylate (EDGMA; Cat. No. 335681, Sigma-Aldrich), trimethoxysilane (PMA; Sigma-Aldrich), dimethyl sulfoxide (DMSO; Sigma-Aldrich), N-hydroxysuccinimide (NHS; Sigma-Aldrich), 1-ethyl-3-(3-dimethylaminopropyl) carbodiimide (EDC; Sigma-Aldrich), potassium persulfate (KPS; Sigma-Aldrich), and polyvinyl alcohol (PVA; Sigma-Aldrich) were obtained from a commercial supplier and used without further purification.

The polymeric complex was synthesized via free-radical polymerization of HEMA using EDGMA as the crosslinker and KPS as the initiator in a PVA-stabilized aqueous medium. Prior to polymerization, DOX and QUE were precomplexed to enhance template–monomer interactions. After polymerization, repeated washing cycles with distilled water and ethanol were performed to remove residual monomers and nonspecifically adsorbed molecules. All experiments were performed in triplicate (n = 3) to ensure reproducibility. UV-Vis measurements for release studies were performed using a UV-1800 spectrophotometer (Shimadzu, Kyoto, Japan).

### 2.2. Precomplex formation

To prepare the precomplex, 6 mg of DOX was dissolved in 500 μL of distilled water, and 3 mg of QUE was dissolved in 500 μL of 2-propanol. The solutions were combined in a single vial and 1.62 μL of PMA was added to facilitate interaction within the prepolymerization system. The mixture was stirred at room temperature for 80 min to ensure homogeneity and promote template–functional component interactions.

### 2.3. Synthesis of molecularly imprinted polymeric complex

For polymerization, 275 mg of PVA was dissolved in 25 mL of distilled water, and 19.8 mg of KPS was dissolved separately in 45 mL of distilled water. In the reactor vessel, 11 mg/mL PVA solution, 0.6 mL of HEMA, and 0.3 mL of EDGMA were introduced sequentially. The prepared precomplex was then added to the reaction system. Polymerization was initiated by adding 0.44 mg/mL KPS under argon atmosphere to provide an inert environment and prevent oxygen inhibition. The reaction mixture was incubated in a water bath at 70 °C for 2 h [5,9]. The polymeric structure was formed via free-radical polymerization of HEMA in the presence of a crosslinking agent, resulting in a stable three-dimensional network. The incorporation of DOX and QUE into the polymer matrix is attributed to noncovalent interactions such as hydrogen bonding and physical entrapment within the crosslinked structure. These interactions play a key role in controlling the release behavior of the active components.

### 2.4. Washing and drying of the polymer

Following polymerization, the obtained complex was purified by washing it three times each with distilled water and ethanol to remove unreacted components and nonspecifically adsorbed residues. The suspension was centrifuged at 14,800 rpm (≈21,000 × *g*) for 20 min. The collected precipitates were dried at room temperature and stored for further analysis [5,9].

Nanoparticle yield was calculated based on the ratio of the dried nanoparticle mass obtained after synthesis to the total initial mass of PVA, KPS, HEMA, and EDGMA used in the formulation:


$$\text{Nanoparticle yield }(\%)=\frac{\text{Dried nanoparticle mass}}{\text{Total initial formulation mass}}\times 100$$

### 2.5. Covalent coupling of folic acid

The dried polymer complex (10 mg) was dispersed in 100 μL of DMSO. Carbodiimide activation was performed by adding 2.4 mg of EDC and 4 mg of NHS under continuous stirring for 2 h. The reaction proceeded in the intrinsic reaction medium without additional pH adjustment. Subsequently, 2.65 mg of folic acid was introduced into the activated suspension, briefly sonicated to ensure uniform dispersion, and stirred for an additional 4 h at room temperature. The final folic acid-functionalized polymeric complex was collected and dried at room temperature [10].

### 2.6. Characterization of the polymeric complex

The surface morphology of the synthesized polymeric complex was examined using scanning electron microscopy (SEM). Morphological analysis was carried out using a GeminiSEM 500 scanning electron microscope (ZEISS, Oberkochen, Germany). Fourier transform infrared (FTIR) spectra were recorded using a Spectrum Two FTIR spectrometer (PerkinElmer, Shelton, CT, USA).

### 2.7. Optimization of release conditions

In vitro release studies were conducted using a dialysis membrane system in 0.1 M acetate buffer at pH 5.5 to simulate mildly acidic tumor-associated conditions [11]. A dialysis membrane with a molecular weight cut-off of 12–14 kDa was used in all release experiments.

The polymeric complex was dispersed at a concentration of 1 mg in 4 mL of buffer (0.25 mg/mL). Experiments were performed under continuous magnetic stirring at controlled room temperature (25 ± 2 °C).

Extended release experiments were also conducted for up to 24 h to evaluate the sustained release behavior of the system. At predetermined time points within a 180-min interval, aliquots were withdrawn and analyzed spectrophotometrically at 460 nm (DOX), 372 nm (QUE), and 375 nm (folic acid). At each sampling point, aliquots of 1 mL were withdrawn and replaced with an equal volume of fresh buffer to maintain constant volume and approximate sink conditions. Drug concentrations were calculated using calibration curves derived from standard solutions. All measurements were conducted in triplicate. The selected experimental duration was chosen to evaluate initial diffusion behavior under acidic conditions and to characterize the short-term release dynamics of the system. The release studies were conducted at pH 5.5 to simulate the acidic tumor microenvironment as well as intracellular endosomal and lysosomal conditions, where drug release is expected to occur.

## 3. Results and discussion

### 3.1. Characterization of the polymeric complex

For the calculation of nanoparticle yield, 900 mg of dried nanoparticles were obtained from a total initial formulation mass of approximately 1253.9 mg. Accordingly, the nanoparticle yield was calculated as 71.8% using the following equation:


$$\text{Nanoparticle yield }(\%)=\frac{900}{1253.9}\times 100$$

**Figure 1:**
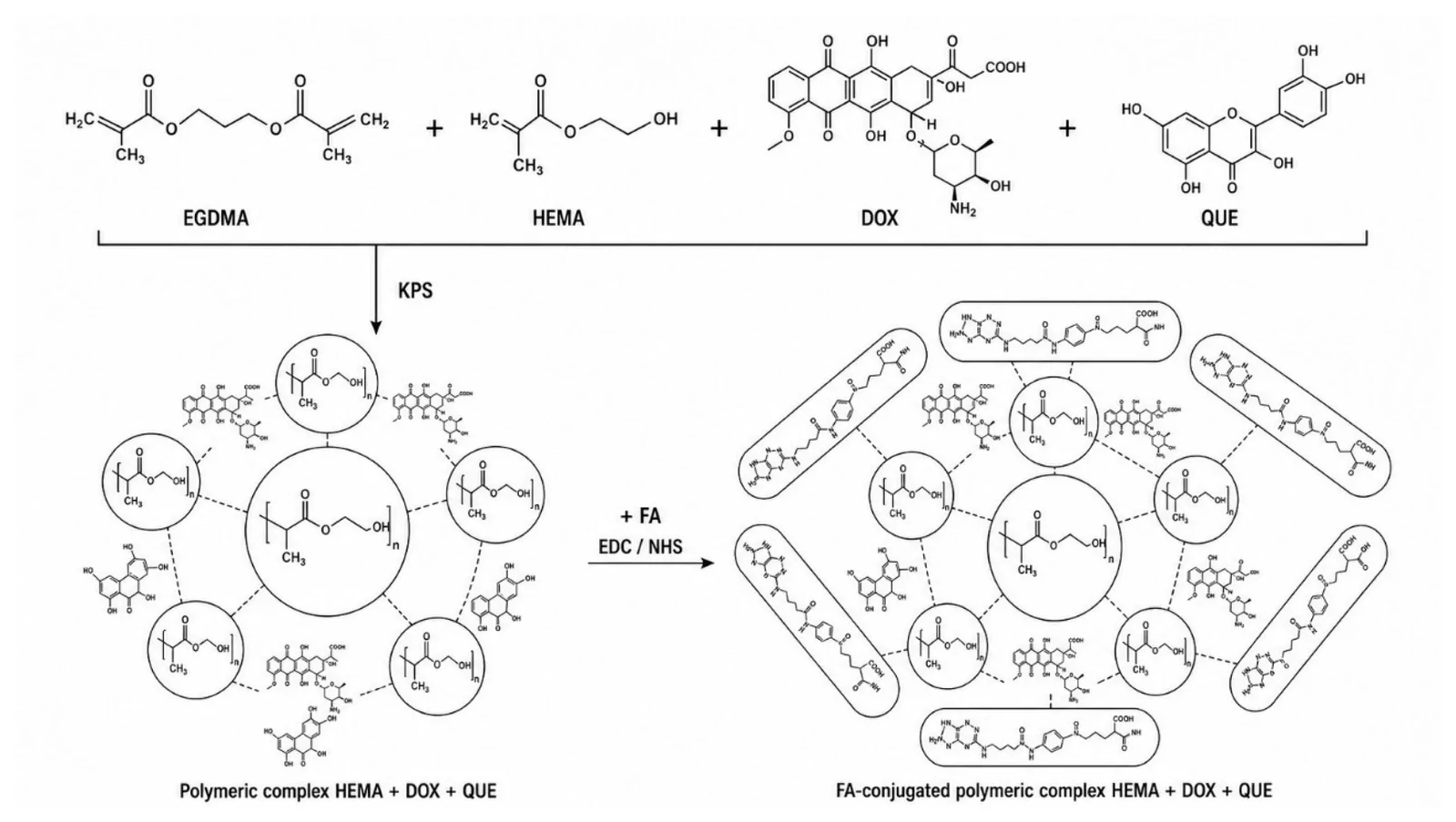
Schematic representation of the formation of the DOX/QUE-loaded poly(HEMA)-based polymeric complex via KPS-initiated free-radical polymerization and subsequent folic acid (FA) conjugation through EDC/NHS chemistry.

**Figure 2:**
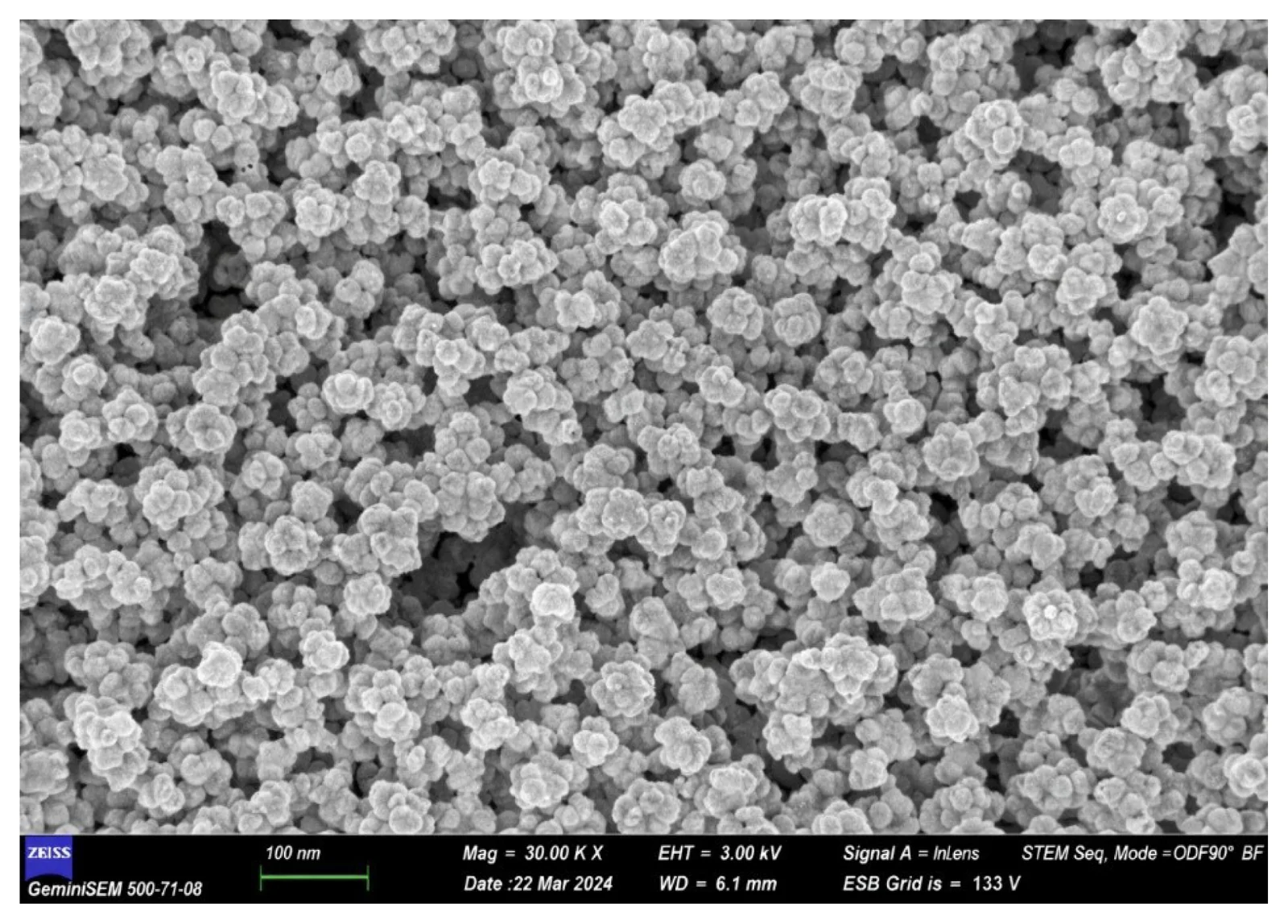
SEM image of the folic acid-functionalized polymeric complex at 30,000× magnification, showing nanoscale spherical morphology with rough surface texture.

SEM micrographs revealed heterogeneous nanoscale structures with irregular and partially aggregated spherical domains. The surface exhibited rough and microporous features, which are likely to enhance effective surface area and facilitate drug loading as well as diffusion-controlled release behavior. Particle size estimation based on scale-bar measurements (n = 30) confirmed nanoscale morphology (Figures 1 and 2).

**Figure 3:**
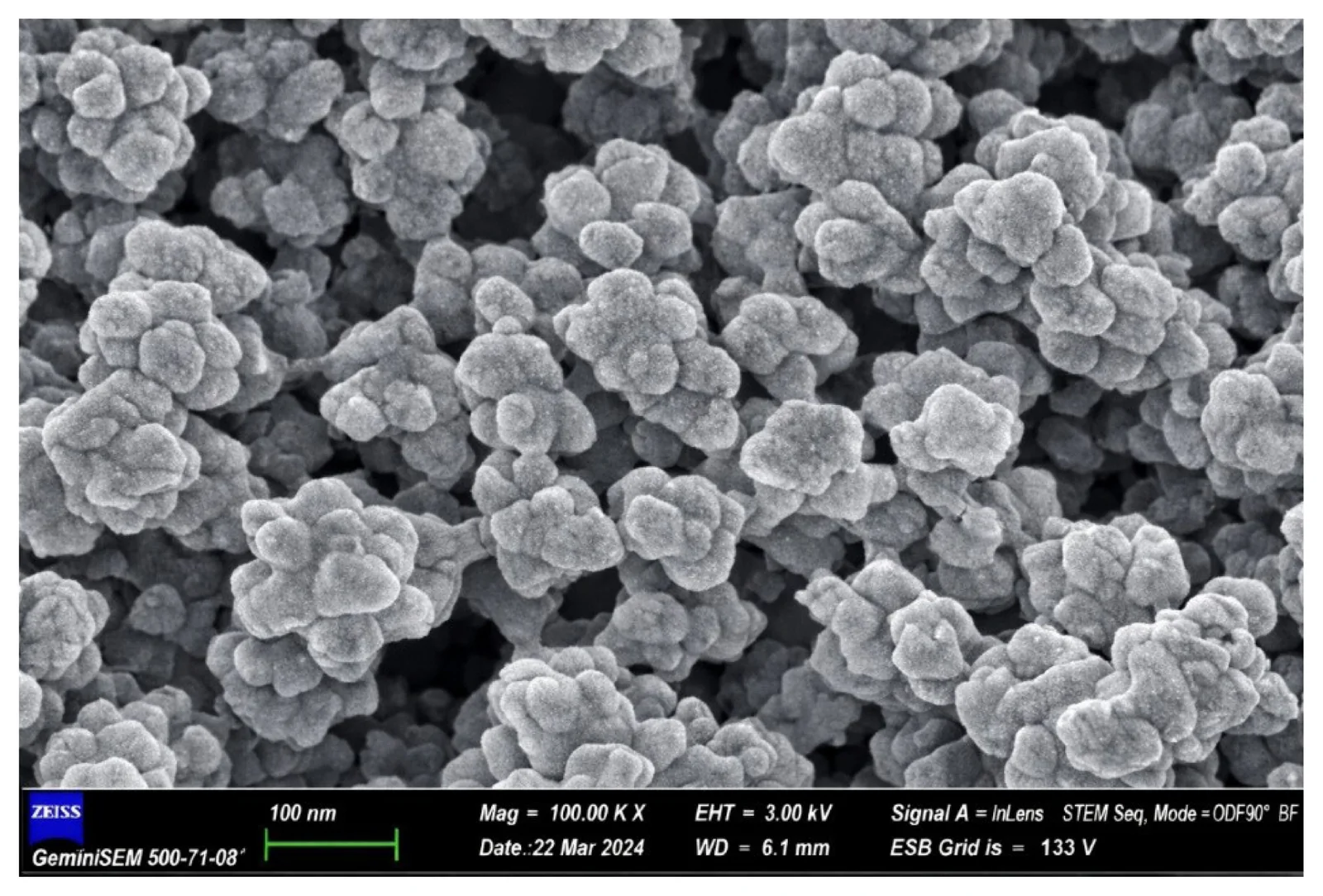
SEM image of the polymeric complex at 100,000× magnification, displaying aggregated nanostructures and rough surface morphology.

SEM images further revealed spherical structures with an average particle size in the range of 60–80 nm, as seen in Figure 3, indicating nanoscale morphology suitable for drug delivery applications.

**Figure 4:**
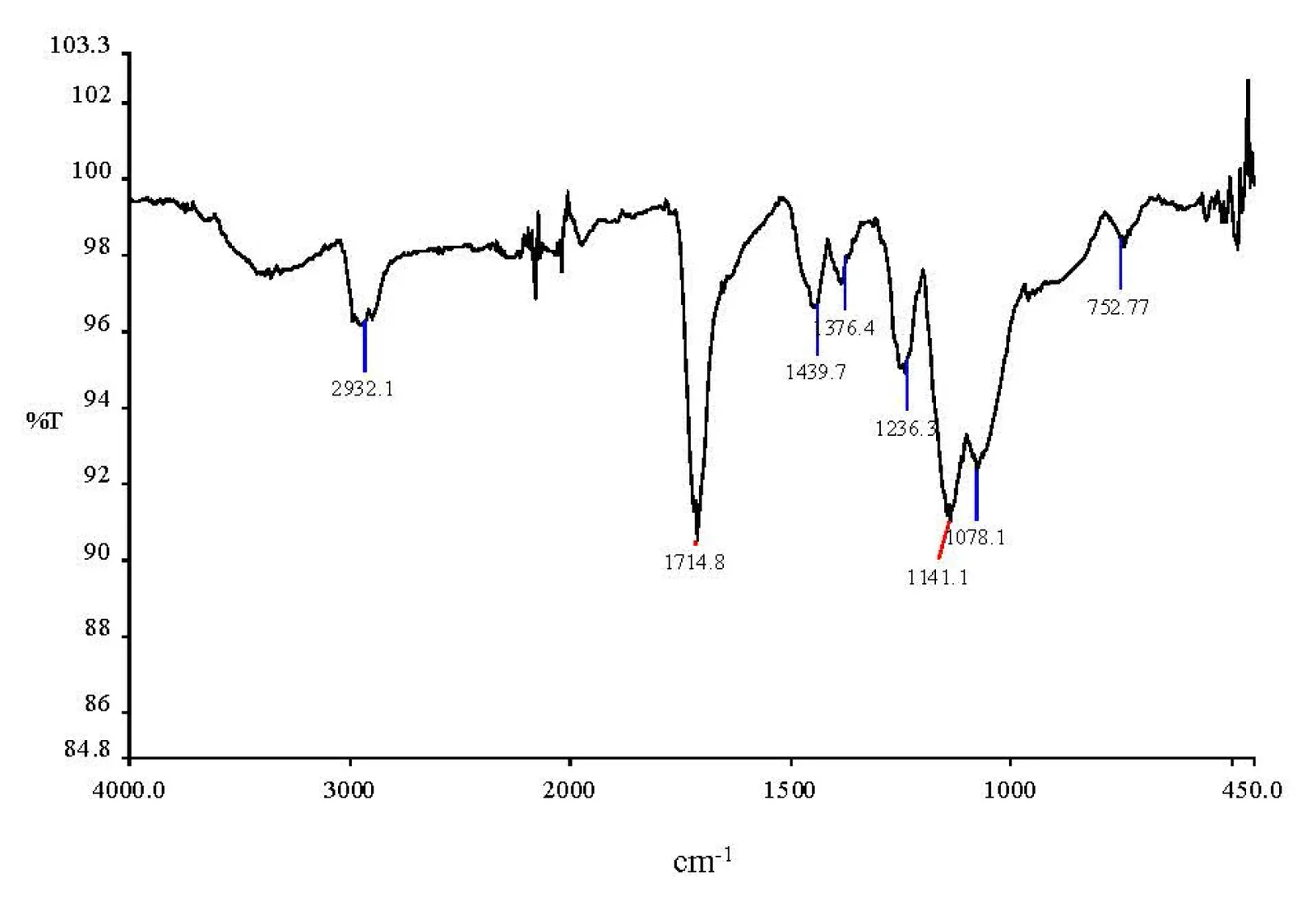
FTIR spectrum of p(HEMA) showing characteristic peaks, including a strong absorption band near 1723 cm^−1^ (C=O stretching).

**Figure 5:**
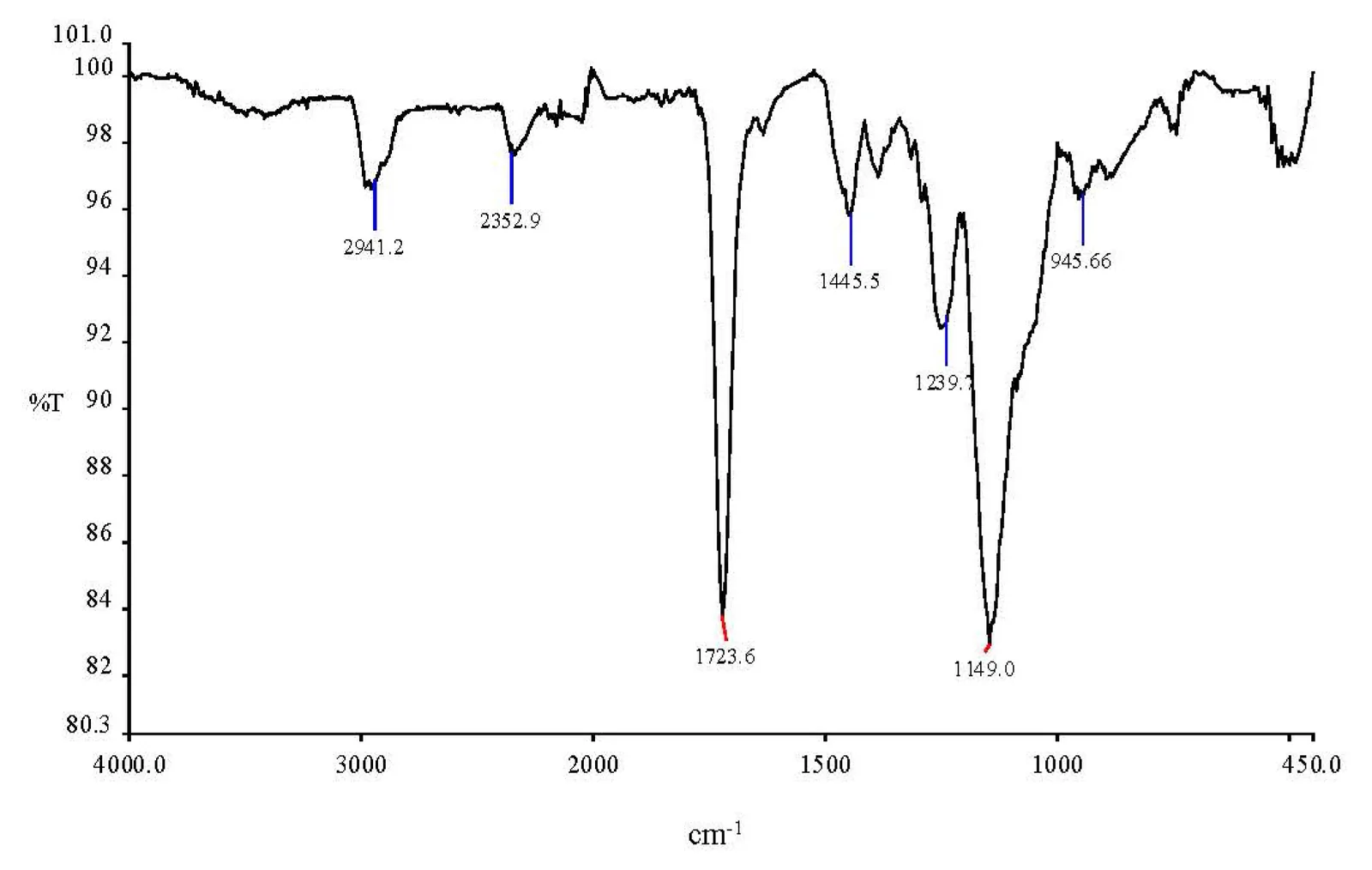
FTIR spectrum of the DOX + QUE imprinted HEMA polymer.

FTIR spectroscopy was employed to verify the chemical structure and successful incorporation of functional components within the polymeric matrix, as seen in Figures 4 and 5.

Characteristic bands corresponding to C=O (1723 cm^−1^) and increased C–H stretching (~1149 cm^−1^) were visible, indicating successful drug loading.

The FTIR spectrum of poly(HEMA) displayed a characteristic C=O stretching vibration near 1720–1723 cm^−1^, corresponding to the ester carbonyl groups of the polymer backbone. Following molecular imprinting with DOX and QUE (DOX + QUE Imp poly(HEMA)), this band was retained, while increased intensity was observed in regions associated with C–O–C and ester-related vibrations (~1149 cm^−1^), indicating the interaction of the loaded molecules with the polymer matrix. After covalent conjugation of folic acid, additional bands were observed at approximately 1147.1 cm^−1^ and 1012.3 cm^−1^. These bands are consistent with functional groups associated with folic acid and support its chemical incorporation into the polymeric structure [5]. Due to the shared poly(HEMA) backbone, partial band overlap was expected; therefore, interpretation was performed based on comparative intensity analysis rather than relying solely on the appearance of entirely new peaks. Although the FTIR spectra of the polymer and loaded systems exhibited similar characteristic peaks, notable differences were observed in peak intensities and slight shifts in specific regions. These variations can be attributed to the incorporation of DOX, QUE, and folic acid into the polymer matrix, indicating interactions between the functional groups of the active components and the polymer structure. FTIR analysis confirmed chemical incorporation and structural modification but did not directly indicate receptor-binding functionality. The presence of folic acid was confirmed by FTIR analysis, but its functional targeting efficiency requires further validation through biological studies.

### 3.2. Evaluation of drug release behavior

**Figure 6:**
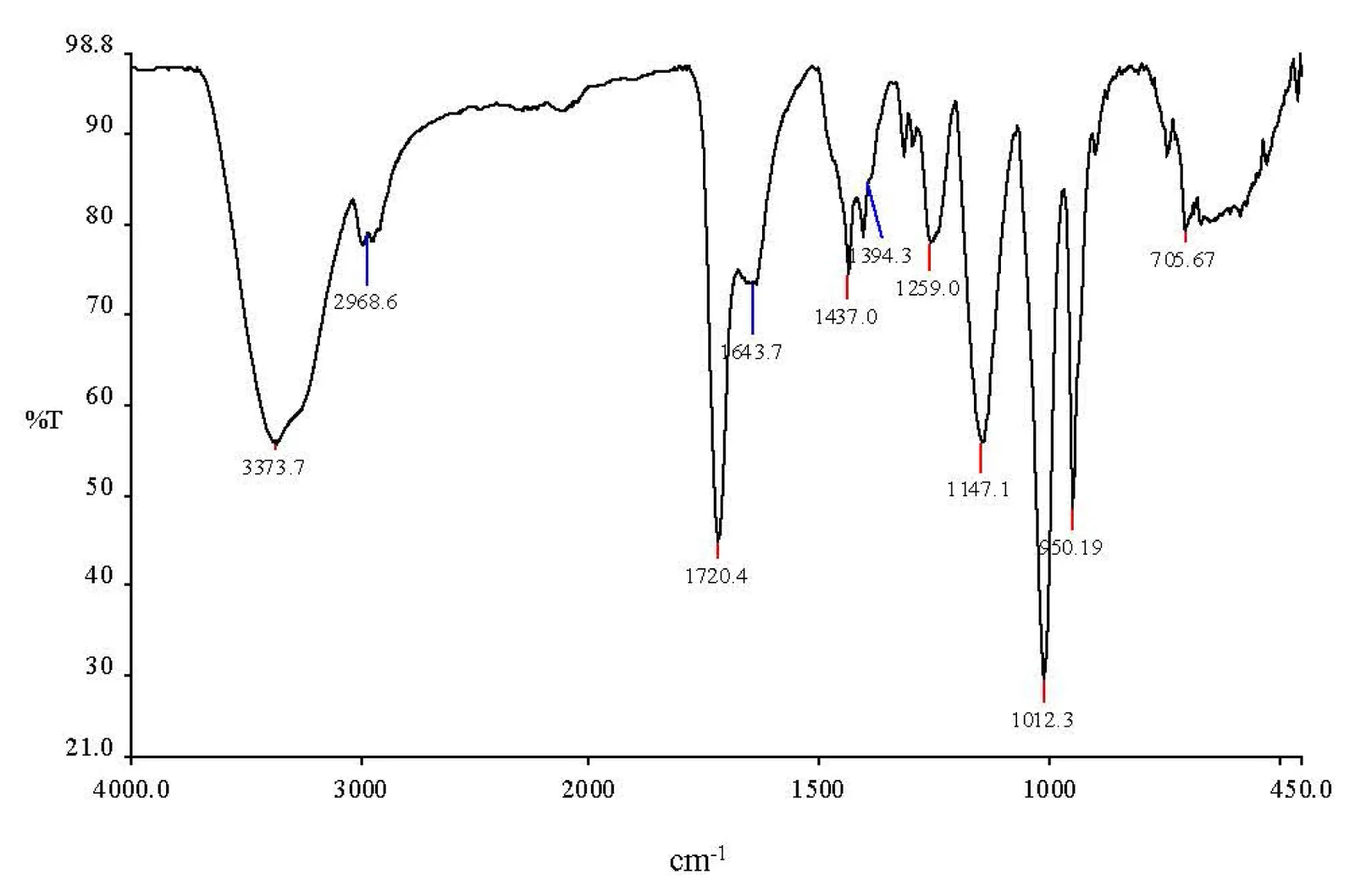
FTIR spectrum of the DOX + QUE imprinted HEMA polymer functionalized with folic acid. New bands at approximately 1147.1 cm^−1^ and 1012.3 cm^−1^ confirm the incorporation of folic acid functional groups.

**Figure 7:**
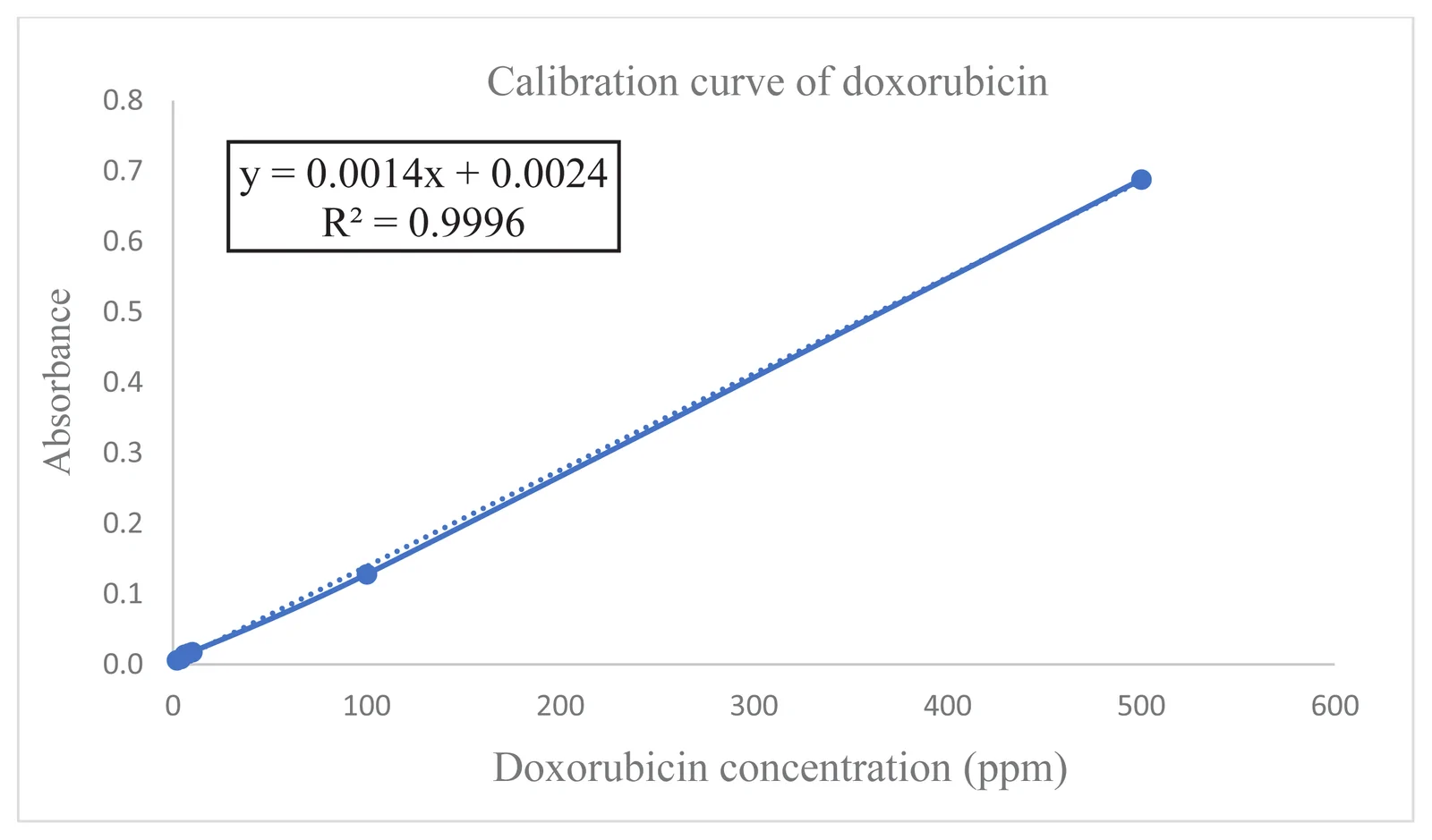
Calibration curve of doxorubicin measured at 460 nm using UV-Vis spectrophotometry. The calibration equation was determined as y = 0.0014× + 0.0024 with a correlation coefficient of R^2^ = 0.9996.

**Figure 8:**
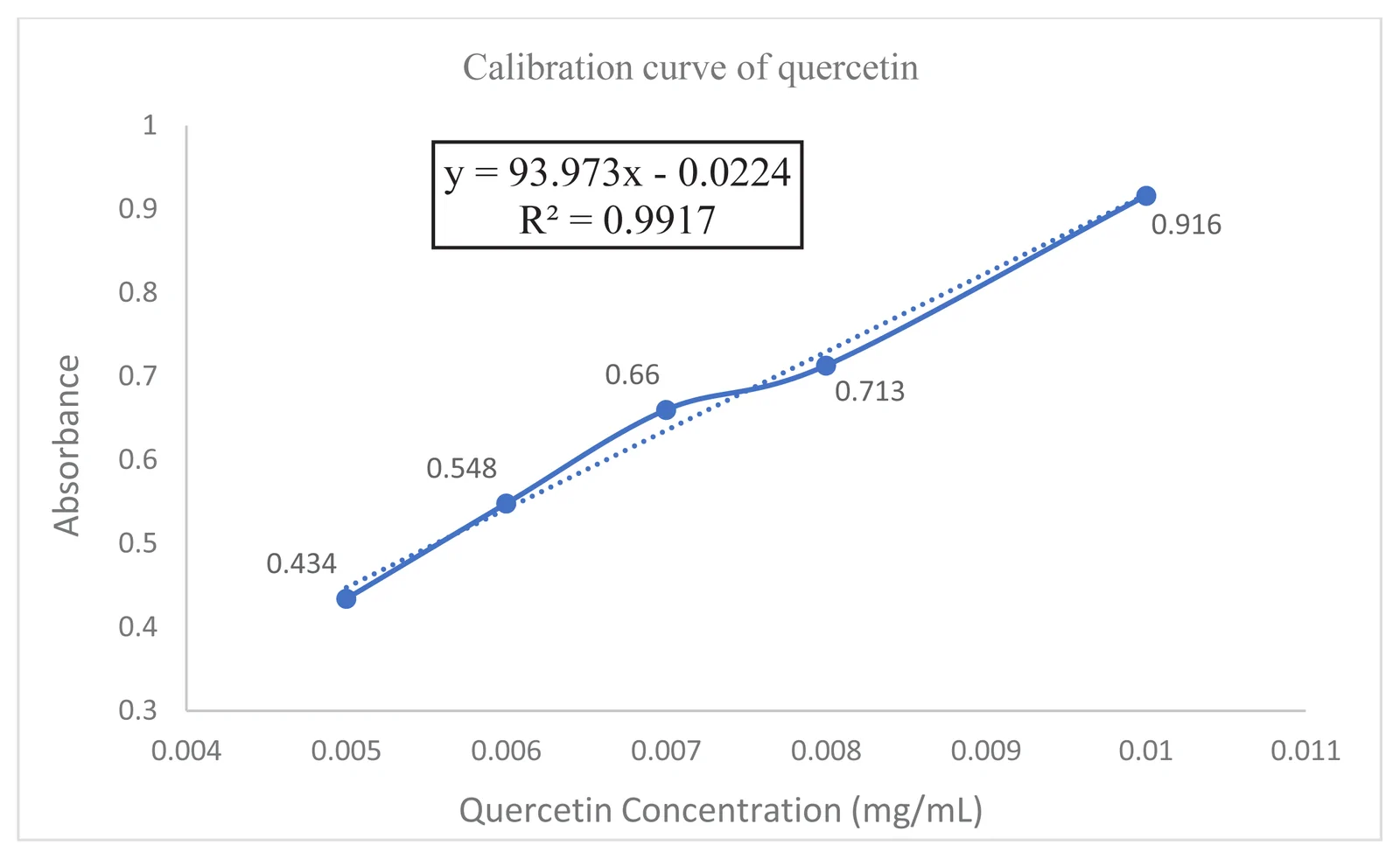
Calibration curve of quercetin measured at 372 nm using UV-Vis spectrophotometry. The calibration equation was determined as y = 93.973× − 0.0224 with a correlation coefficient of R^2^ = 0.9917.

**Figure 9:**
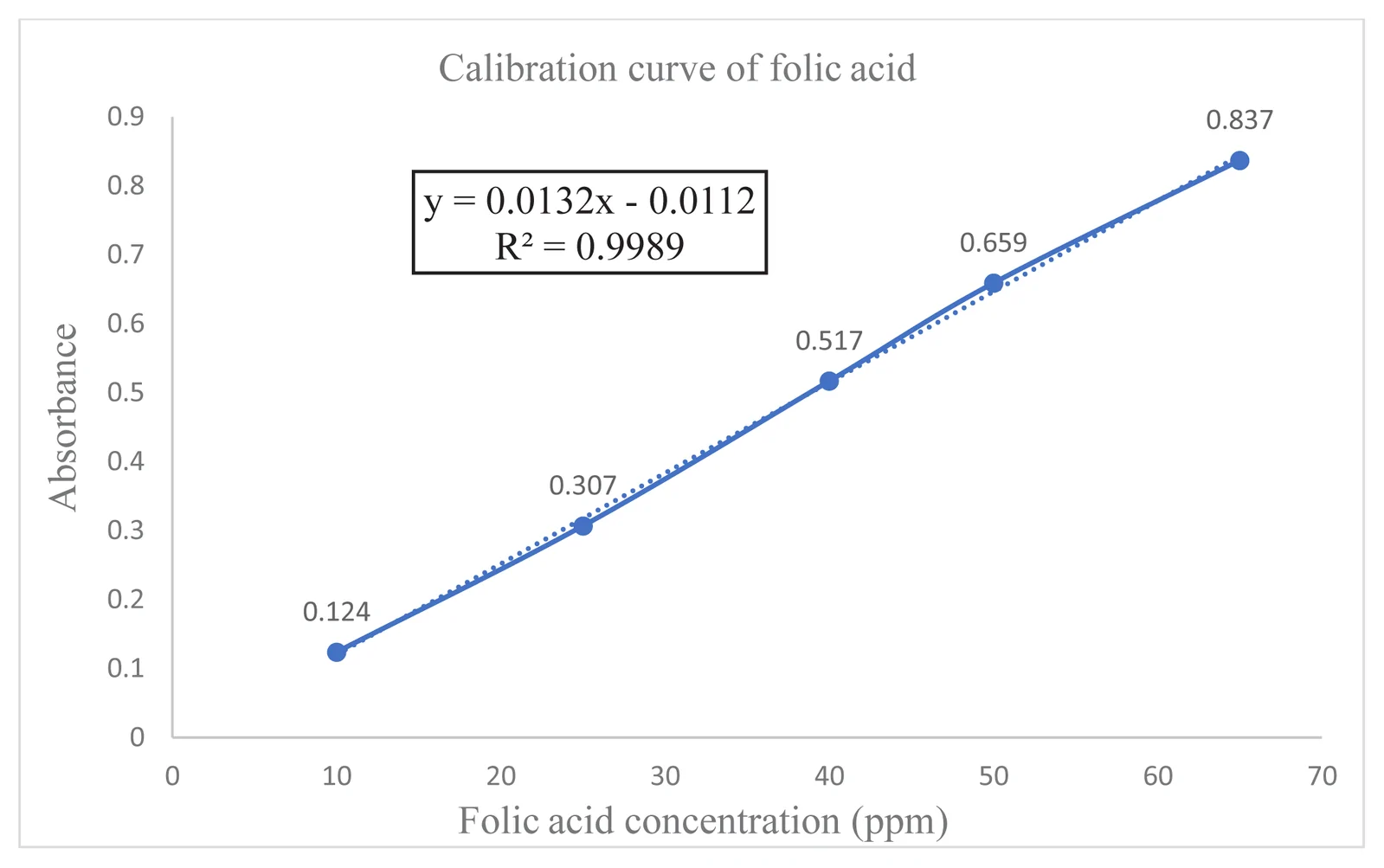
Calibration curve of folic acid measured at 375 nm using UV-Vis spectrophotometry. The calibration equation was determined as y = 0.0132× − 0.0112 with a correlation coefficient of R^2^ = 0.9989.

To evaluate the release performance of the imprinted polymeric complex, calibration curves were established for DOX, QUE, and folic acid using UV-Vis spectrophotometry. Absorbance measurements were performed at 460 nm (DOX), 372 nm (QUE), and 375 nm (folic acid). Linear regression equations derived from standard solutions were used to calculate drug concentrations during the release experiments [1,6,8] (Figures 6–9; Table 1).

The absorbance values presented in Table 1 confirm the measurable presence of the drug within the dialysis membrane after the release period. These values were used to determine the corresponding concentrations with the help of the established calibration equations. The absorbance values measured for the compounds indicated that DOX had values of 0.051 and 0.063 at 460 nm, while folic acid had values of 0.109 and 0.123 at 375 nm. QUE demonstrated comparatively higher absorbance values of 0.144 and 0.145 at 372 nm. These results suggest differences in the residual concentrations of the compounds within the dialysis membrane, reflecting their distinct diffusion and interaction behaviors within the polymeric system.

**Figure 10:**
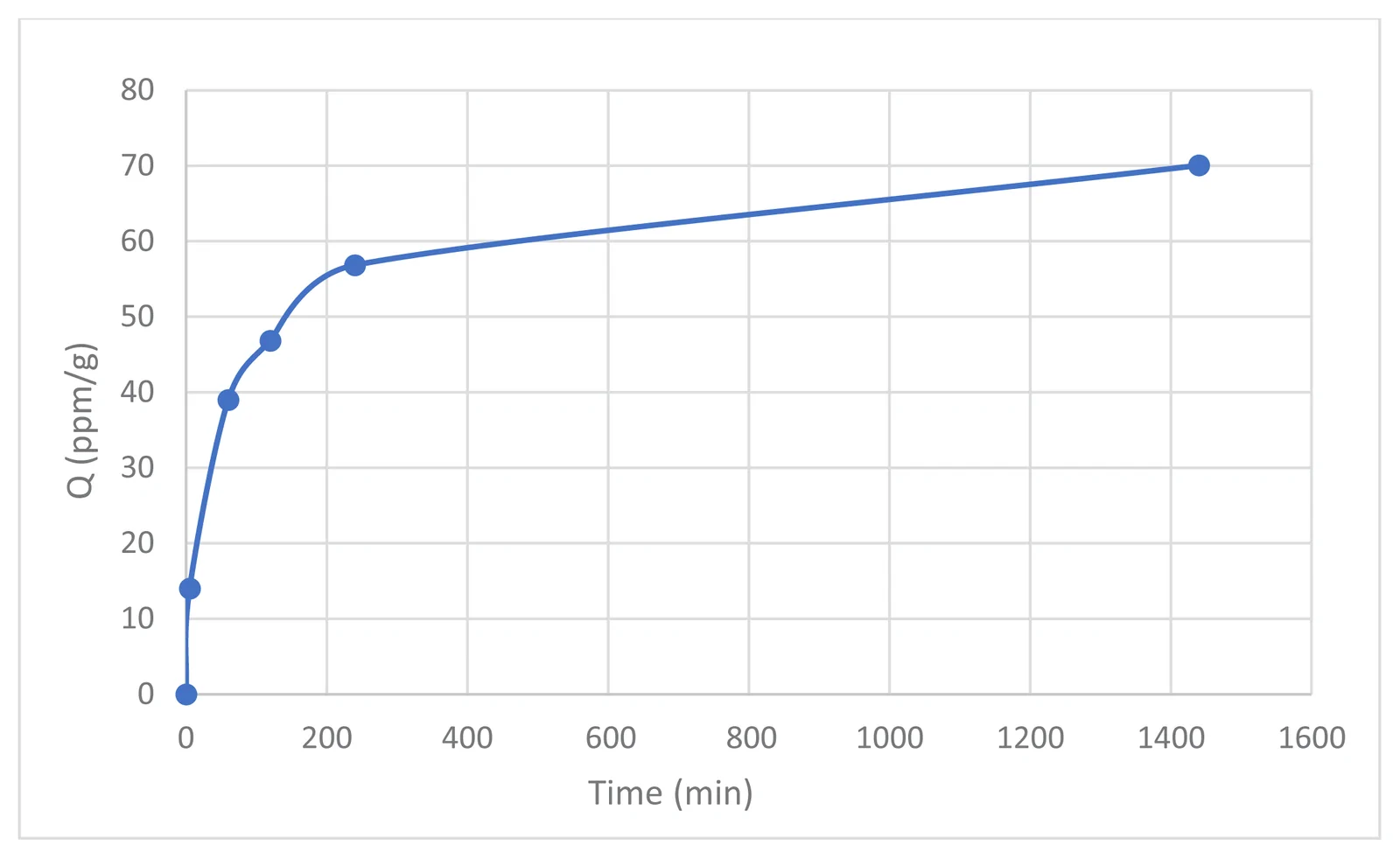
Release profile (Q, ppm/g) of the polymeric system over 24 h

Following the initial release observed within the first 180 min, the polymer exhibited a slower and controlled release profile over the extended duration of the experiment. These results confirm the stability of the polymer matrix and its ability to provide sustained release beyond the initial phase (Figure 10). Initial rapid release within the first 180 min was followed by a sustained and controlled release phase, indicating the stability of the polymer matrix.

Release experiments were conducted using a dialysis membrane system in 0.1 M acetate buffer at pH 5.5. The polymeric complex was incubated for 180 min under continuous stirring at 25 ± 2 °C. Drug release was calculated using a mass-balance approach based on the difference between the initially loaded amount and the remaining amount inside the membrane (Table 2).

DOX exhibited partial release, with 0.0011 mg released from an initial loading of 0.00795 mg, corresponding to approximately 13.8% release within the experimental timeframe. In contrast, QUE demonstrated substantially higher diffusional release, with 0.0978 mg released from an initial 0.0985 mg.

Folic acid was covalently conjugated to the polymer backbone and was therefore not expected to undergo significant diffusion. Its quantification was performed primarily to verify structural presence rather than release functionality [5].

The observed release behavior reflects short-term diffusion characteristics under mildly acidic conditions and demonstrates the differential interaction of the incorporated agents with the imprinted polymer matrix.

### 3.3. Release kinetic analysis text

Release kinetic analysis was performed using the cumulative release data obtained at 0, 5, 15, 60, 120, 240, and 1440 min. The cumulative release amount increased rapidly during the initial stage, reaching approximately 39 ppm/g within the first 15 min, followed by a slower and sustained release phase. The maximum release amount was observed as approximately 70 ppm/g at 1440 min.

The release data were fitted to zero-order, first-order, Higuchi, and Korsmeyer–Peppas kinetic models. The calculated correlation coefficient values indicated that the Korsmeyer–Peppas model provided the best fit among the tested models, with an R^2^ value of approximately 0.808. The release exponent, n, was calculated as 0.334, suggesting that the release behavior was mainly governed by diffusion-controlled release. All calculation data are provided in Table 3.

The cumulative release profile demonstrated biphasic release behavior, characterized by an initial burst release followed by a slower sustained release phase. The release amount reached approximately 39 ppm/g within the first 15 min and increased gradually to 70 ppm/g after 1440 min. To evaluate the release mechanism, the experimental data were fitted to zero-order, first-order, Higuchi, and Korsmeyer–Peppas kinetic models. Among these models, the Korsmeyer–Peppas model showed the highest correlation coefficient value (R^2^ = 0.808). The release exponent, n, was calculated as 0.334, indicating a predominantly diffusion-controlled release mechanism.

### 3.4. Encapsulation efficiency and drug loading capacity

Encapsulation efficiency and drug loading capacity values for DOX and QUE were recalculated based on the experimentally determined drug amounts within the polymeric system. Encapsulation efficiency (EE) was calculated using the following equation:


EE(%)=(Amount of encapsulated drug/Initial amount of drug used)×100

For DOX, the initial amount used was 6 mg and the amount detected within the polymeric system was 0.00795 mg. Therefore, the encapsulation efficiency of DOX was calculated as follows:


EEDOX=(0.00795/6)×100=0.1325%

For QUE, the initial amount used was 3 mg and the amount detected within the polymeric system was 0.0985 mg. Therefore, the encapsulation efficiency of QUE was calculated as follows:


EEQUE=(0.0985/3)×100=3.28%

In addition to encapsulation efficiency, the drug loading capacity of the polymeric system was evaluated using the following Q-value calculation:


Q (mg/g)=Amount of drug in the polymeric system (mg)/Amount of polymer (g)

Since 1 mg of polymeric complex (0.001 g) was used in the release experiments, the calculated drug loading capacities were determined as follows:


$$\begin{array}{l} \text{QDOX}=0.00795\;\text{mg}/0.001\;\text{g}=7.95\;\text{mg}/\text{g}\\ \text{QQUE}=0.0985\;\text{mg}/0.001\;\text{g}=98.5\;\text{mg}/\text{g} \end{array}$$

These calculated values allow for quantitative evaluation of both encapsulation efficiency and drug loading behavior of the polymeric complex.

### 3.5. Interpretation of release performance

The differential release behavior suggests stronger interactions between DOX and the imprinted polymer matrix compared to QUE. This may be attributed to differences in molecular size, hydrophobicity, and possible hydrogen-bonding interactions within the crosslinked poly(HEMA) network. The relatively rapid diffusion of QUE may indicate weaker retention within the polymer structure under the selected experimental conditions.

The folic acid-functionalized surface is intended to provide receptor-associated targeting capability based on established folate receptor interactions reported in the literature [8]. However, functional targeting efficiency was not directly evaluated in the present study.

Overall, the system demonstrates controlled and differential diffusion behavior at the material level without implying clinical performance.

The release behavior suggests a diffusion-controlled mechanism during the initial phase, followed by a more sustained and matrix-controlled release pattern. The comparatively higher release of QUE may be attributed to weaker interactions with the polymer matrix, whereas DOX shows stronger retention due to more stable interactions within the imprinted sites.

## 4. Conclusion

A folic acid-functionalized, DOX + QUE imprinted poly(HEMA)-based polymeric complex was successfully synthesized and structurally characterized in this study. FTIR analysis confirmed the preservation of the polymer backbone together with the incorporation of DOX, QUE, and folic acid, while SEM imaging revealed a nanoscale morphology with partially aggregated spherical domains.

In vitro release experiments conducted under mildly acidic conditions (pH 5.5) demonstrated differential release behavior of the incorporated agents. DOX exhibited partial retention within the imprinted polymer matrix, whereas QUE showed comparatively higher diffusional release. These findings suggest selective interactions between the active components and the crosslinked polymer network, consistent with imprinting-related binding characteristics.

The folic acid-functionalized structure provides a potential targeting feature based on known folate receptor interactions reported in the literature. However, its functional targeting efficiency requires further biological validation.

Overall, the developed dual-drug imprinted polymeric system demonstrates physicochemical feasibility for differential and sustained release under acidic conditions and provides a structurally validated platform for future functional and biological investigations.

## Figures and Tables

**Table 1:** Residual drug concentrations inside the dialysis membrane based on UV absorbance measurements.

Residual drug concentration inside the dialysis membrane						
Chemical (wavelength, nm)	Doxorubicin (460)		Folic acid (375)		Quercetin (372)	
Absorbance values	0.051	0.063	0.109	0.123	0.144	0.145

**Table 2:** Drug loading and release amounts within the dialysis membrane.

	Drug content inside the dialysis membrane		
Chemical	Initial amount (mg)	Remaining (mg)	Released (mg)
Doxorubicin	0.00795	0.00685	0.0011
Quercetin	0.0985	0.0007	0.0978

**Table 3:** Kinetic models.

Kinetic model	Linearized equation	Kinetic constant	R^2^
Zero-order	Qt = k_0_t + C	k_0_ = 0.0296 ppm/g min^−1^	0.382
First-order	ln Q∞ − Qt = ln Q∞ − k_1_t	k_1_ = 0.00638 min^−1^	0.728
Higuchi	Qt = kH t^1/2^ + C	kH = 1.528 ppm/g min^−1/2^	0.622
Korsmeyer–Peppas	log Mt/M∞ = log k + n log t	n = 0.334; k = 0.157	0.808

## Data Availability

All data generated or analyzed during this study are included in this article.
